# Mottling score is a strong predictor of 14-day mortality in septic patients whatever vasopressor doses and other tissue perfusion parameters

**DOI:** 10.1186/s13054-019-2496-4

**Published:** 2019-06-10

**Authors:** Guillaume Dumas, Jean-Rémi Lavillegrand, Jérémie Joffre, Naïke Bigé, Edmilson Bastos de-Moura, Jean-Luc Baudel, Sylvie Chevret, Bertrand Guidet, Eric Maury, Fabio Amorim, Hafid Ait-Oufella

**Affiliations:** 1 Assistance Publique – Hôpitaux de Paris (AP-HP), Hôpital Saint-Antoine, Service de réanimation médicale, 184 rue du Faubourg Saint-Antoine, 75571 Paris, cedex 12 France; 2 Sorbonne Université, Paris, France; 3 ECSTRA team, Biostatistics and clinical epidemiology, UMR 1153 (center of epidemiology and biostatistic Sorbonne Paris Cité, CRESS), INSERM, Paris Diderot University, Paris, France; 4 Adult Intensive Care Unit, Hospital Santa Luzia, School of Medicine, Brasília, Brazil; 5 Inserm U1136, F-75012 Paris, France; 6 Université de Paris, Inserm U970, Centre de Recherche Cardiovasculaire de Paris (PARCC), Paris, France

**Keywords:** Mottling, Septic shock, Microcirculation, Outcome, Vasopressor

## Abstract

**Background:**

Mottling score, a tissue perfusion parameter, is correlated with outcome in septic shock patients. However, its predictive value on mortality according to prognostic covariates such as vasopressor dose and other tissue perfusion parameters remains unknown.

**Methods:**

Mottling score and tissue perfusion parameters were recorded at ICU admission (H0), H-6, H 12, and H-24 and used to assess the predictive value of mottling score on 14-day mortality in a development cohort. Results were then validated in an independent cohort of septic shock patients in Brazil.

**Results:**

Overall, 259 patients with sepsis or septic shock were included, 14-day mortality was 37%. Factors associated with death were mottling score (OR 2.26 [95% CI, 1.72–2.97]), arterial lactate level (OR 1.29 [1.11–1.5]), and urine output < 0.5 ml/Kg/h (OR 3.03 [1.37–6.69]). The C statistic for the model was 0.90 in the development cohort and 0.76 in the validation cohort. The predictive value of mottling score was not affected by vasopressor doses (*p* for interaction = 0.33): OR for mottling score ranged from 2.34 [1.10–3.15] in patients without vasopressor to 3.84 [1.98–7.43] in patients infused with high doses of vasopressor (> 0.8 μg/kg/min). There was no difference in the effect of mottling score on mortality according to mean arterial pressure, heart rate, cardiac index, and urine output, but we found a significant interaction between arterial lactate level and mottling score (*p* = 0.04). The predictive value of the mottling score remains significant when using the recent SEPSIS-3 definition of septic shock. Finally, a decrease of mottling score during resuscitation was significantly associated with better outcome after adjustment on SOFA score (*p* = 0.001).

**Conclusions:**

Our results support the high prognostic value of mottling score for 14-day mortality in septic patients, whatever vasopressor dosage and other perfusion parameters. Mottling score variations during resuscitation are also predictive of mortality.

**Electronic supplementary material:**

The online version of this article (10.1186/s13054-019-2496-4) contains supplementary material, which is available to authorized users.

## Background

Sepsis is a common reason for intensive care unit (ICU) admission, responsible for high morbidity and mortality [1, 2]. Alongside macrohemodynamic parameters abnormalities, microcirculation blood flow impairment has been identified in septic patients [3–5], being more pronounced in the most severely ill patients [6]. These microvascular disorders have been associated with mortality [4] as well as their persistence despite resuscitation [7]. These abnormalities could persist even if macro-hemodynamics parameters have been normalized, suggesting a dissociation between micro and macrocirculatory compartments in a number of patients [8–10].

Therefore, identifying and monitoring microcirculatory perfusion alterations could be of interest. Assessing regional perfusion in sublingual area or gastric mucosa has been validated but remains difficult to use at the bedside. Peripheral and more specifically skin perfusion is more easily to assess. Mottling, defined as patchy skin discoloration, is a common sign of cutaneous hypoperfusion [9, 11]. Blood flow reduction may be due to local vasoconstriction and endothelial dysfunction [9, 12]. Recently, Brunauer et al. [13] reported in septic shock patients a significant correlation between skin mottling extension and kidney perfusion (assessed by the pulsatility index), supporting the concept that mottling reflects global tissue hypoperfusion. Our group has developed a clinical score, based on the extension of mottling around the knee (ranging from 0 to 5), with very good inter-observer agreement [14]. It has been previously found that mottling score, measured 6 h after initial resuscitation in ICU, is a strong predictor of mortality in patients with septic shock at day 14 [14] and at day 28 [15].

However, several issues are still into debate. First, it is unclear if skin microcirculation alterations can predict mortality additionally to the main prognostic features of septic shock patients. Second, does vasopressor dosage impact on predictive value of mottling score for mortality? Finally, do mottling score variations during resuscitation predict outcome? These questions are of importance, because mottling, an easy to assess clinical sign, would be an interesting tool to alert and guide management.

To address these clinical questions and assist decision-making, we conducted a study on a large cohort of patients with sepsis or septic shock. The validity of the results was then tested in a separate cohort of patients.

## Methods

### Study design and patients

The patients included in the study had previously been included in studies conducted by our group between January 1, 2011, and December 31, 2016, with the appropriate ethics committee approval [14, 16–18]. Data of all patients with sepsis or septic shock were reviewed, and medical record was consulted if necessary. Each patient was included once. Sepsis was defined according to international consensus definitions [19]. Patients were admitted from the emergency department or the medical wards. Patients with sepsis were included at ICU admission; patients with septic shock were included when vasopressors were required (within 3 h of admission). Patients were excluded if mottling score was not available at inclusion and in case of dark skin. Because we focused on parameters recorded 6 h after inclusion (H6), patients who died before H6 were also excluded. Data from these patients, who are referred to as the development cohort, were used to develop the main analysis.

Next, our analysis was validated in an independent cohort of patients with septic shock who were admitted in a mixed medical/surgical adult ICU in Brazil [15]. Patients had been prospectively enrolled in 2012–2013 and screened according to the same inclusion and exclusion criteria as those used for the development cohort. This group of patients is referred to as the validation cohort. The same clinical and laboratory data were available for analysis in both cohorts, with the exception of repeated measures every 6 h available in the development cohort only. The flow chart of the study is displayed in Additional file 1: Figure S1.

### General management

Management of patients was guided by our local protocol, adapted from international guidelines [20]. Treatment was standardized including volume expansion and if necessary vasopressors (norepinephrine or epinephrine) used in a stepwise manner to achieve pre-defined endpoints: mean arterial pressure (MAP) ≥ 65 mmHg and urinary output ≥ 0.5 ml/kg/h.

All patients were investigated with transthoracic echocardiography (Vivid 7 Dimension’06, GE, Healthcare®) to evaluate left ventricular function, volume status, and cardiac output. When a cardiac dysfunction (left ejection fraction < 30% by Simpson’s biplane methodology) was identified, an inotropic therapy was introduced and/or epinephrine replaced norepinephrine. Ventilation support was provided when needed. If required, patients were sedated with propofol and/or midazolam and analgesia provided with sufentanil. Use of low doses hydrocortisone (200 mg/day) was considered when there was persistence of high dosage of vasopressors requirement despite a perceived adequate intravascular volume.

### Data collection

Data reported in Table 1 were prospectively collected. Baseline information was recorded at ICU admission, including severity of illness evaluated by the Sequential Organ Failure Assessment (SOFA) score (within 6 h of admission and H24) and Simplified Acute Physiology Score II (SAPS II). Hemodynamic variables were recorded at inclusion (H0) and at H6, H12, and H24. We measured MAP, heart rate (HR), diuresis, cardiac index using echocardiography, arterial lactate level, and mottling score. Mottling score provided a semi quantitative evaluation of mottling based on skin area extension on legs: Score 0 no mottling, score 1 small mottling area (coin size) localized to the center of the knee, score 2 mottling area that does not exceed the superior edge of the knee cap, score 3 mottling area that does not exceed the middle thigh, score 4 mottling area that does not exceed the fold of the groin, and score 5 otherwise. Vasopressor doses were recorded every 6 h. Because different agents were used, we transformed epinephrine in norepinephrine equivalent [4] and all doses being expressed in μg/kg/min.

**Table 1:** Characteristics of 259 critically ill patients with sepsis or septic shock

Demographic data	*N* (%) or Median [IQR]	
Age, year	68 [57–80]	
Male sex	133 (51)	
Body-mass index, kg/m^2^	25 [22–28]	
SAPS II score, points	54 [41–70]	
SOFA score at day 1, points	10 [5–14]	
Pre-existing conditions		
Atherosclerotic disease	67 (26)	
Cirrhosis	27 (12)	
Sepsis origin		
Community-acquired pneumonia	113 (44)	
Intra-abdominal	65 (26)	
Urinary tract	28 (11)	
Skin and soft tissue	21 (8)	
Others	27 (11)	
Clinical parameters	At H0	At H6
Heart rate, beats/min	105 [89–121]	100 [85–118]
Mean arterial pressure, mm Hg	71 [63–80]	73 [67–82]
Cardiac index, l/min/m^2^	4.9 [3.9–6.2]	5.0 [3.9–6.03]
Urinary output, ml/kg/h	–	0.46 [0.13–0.90]
Arterial lactate, mmol/l	2.8 [1.5–5.6]	2.4 [1.4–5.3]
Mottling score		
0	109 (42)	125 (49)
1	22 (8)	36 (14)
2	51 (20)	29 (11)
3	41 (16)	29 (11)
4	16 (6)	14 (5)
5	20 (8)	24 (9)
Drugs		
Norepinephrine	156 (60)	188 (73)
Doses, μg/kg/min	0.4 [0.2–0.7]	0.5 [0.2–1]
Epinephrine	13 (5)	13 (5)
Doses, μg/kg/min	0.5 [0.2–0.6]	0.6 [0.3–1]
Dobutamine	3 (1)	4 (2)
Doses, μg/kg/min	0.4 [0.3–0.75]	0.3 [0.18–1.3]
Mechanical ventilation at day 1	127 (49)	
Mortality at day 14	95 (37)	

Results are given as *N* (%) or median [IQR]

Simplified Acute Physiology Score (SAPS II) was calculated within 24 h of intensive care unit admission. Sequential Organ Failure Assessment (SOFA) score was calculated within 24 h of septic shock onset. *MAP* mean arterial pressure

The main outcome was the 14-day mortality.

### Statistical analysis

Continuous variables are described as median and interquartile range (IQR) and compared using Wilcoxon’s rank sum test; categorical variables are summarized by counts (percents) and compared using exact Fisher test.

First, in the development cohort, hemodynamics (MAP, HR, cardiac index) values, global perfusion parameters (i.e., arterial lactates), organ-specific tissue perfusion parameters (mottling score, urine output) recorded at H-6, and patients’ characteristics were tested in univariate analyses for association with 14-day mortality. In those models, mottling score and biological parameters were expressed continuously, after checking their log linear relationship with the outcome using restricted cubic splines with equally space knots (10th, 50th, and 90th) (Additional file 1: Figures S2 and S3).

Thereafter, all factors achieving *P* < 0.10 in univariate analyses were entered into the multivariate logistic regression model. A multiple backward-stepwise selection procedure eliminated those variables with an exit threshold set at *P* = 0.05, after testing for collinearity between variables and checking the assumption of log-linearity as above.

The two core models were as follows. In model 1, continuous variables for which this assumption was violated (vasopressor dose, urine output, MAP, HR) were introduced as binary variables according to threshold of clinical significance. In model 2, these variables were introduced as spline functions with varying number of knots and model selection based on the AIC [21].

In both models, calibration was assessed by the Hosmer–Lemeshow goodness of fit and discrimination by the C statistic. Internal validity was estimated with bootstrap procedure on 200 samples [21, 22]. We then assessed both discrimination and calibration in the independent validation sample. Both were compared to that of the SOFA score.

We looked for interactions of the mottling score effect on mortality, adjusted on SOFA score, with 6 pre-specified characteristics (vasopressor dose, MAP, HR, cardiac index, urine output, arterial lactates), using Gail and Simon test [23]. For the sake of clarity, subgroups were defined by thresholds of clinical significance (MAP, HR, cardiac index, urine output) or based on recent SEPSIS-3 definition [24] for arterial lactates with a cut-off at 2 mmol/l). Vasopressor dose was also considered as a continuous variable, using restricted cubic splines with 3 knots at 10th, 50th, and 90th percentiles (corresponding to 0, 0.3, 1.5 μg/kg/min).

We also studied the effect (adjusted on SOFA score) on mortality of the mottling score variation assessed between admission and after the 6 first hours of resuscitation.

Primary analyses were performed on complete cases after imputation of missing data; actually, to handle missing data on covariates, multiple imputations with chained equation, based on *M* = 30 imputed complete datasets [25], were used. Note that mottling score and arterial lactate had no missing data.

We assessed the sensitivity of our findings by repeating the primary analysis under varying assumptions about the study population. First, we studied our model in the subset of patients with vasopressor only, whatever the type and after exclusion of patients who received epinephrine. Second, we identified patients fulfilling the recent SEPSIS-3 definition of septic shock. We then compared clinical characteristics and outcome according to these criteria and evaluate the performance of our model in this subset. Third, we evaluate the discrimination of our model to predict in-ICU and in-Hospital mortality. Last, analyses were reran on the complete cases (free of imputation).

The measures of associations are presented with odds ratios and 95% confidence intervals. All tests were two-sided, and *p* values lower than 5% were considered to indicate significant associations. Analyses were performed using R statistical platform, version 3.0.2 (https://cran.r-project.org/).

## Results

### Studied population

Overall, 259 patients (age, 68 [57–80] years, 51% male, SAPS II, 54 [41–70]) were included in the development cohort and 97 patients (age, 77 [69–84] years, 43% male, SAPSII 46 [36–57]) in the validation cohort **(**Additional file 1: Figure S1 and Table S1).

Table 1 reports the main characteristics at ICU admission and the hemodynamic parameters at H0 and H6. At H6, 201 (77%) patients were still infused with vasopressor (mainly norepinephrine, *n* = 188 (73%), median dose of 0.5 [0.2–1] μg/kg/min). Forty-nine percent (*n* = 127) of the patients required invasive mechanical ventilation. Median SOFA score was 10 [5–14]. The most common sepsis etiology was pneumonia (*n* = 113, 44%). Distribution of mottling score is detailed in Table 1.

### Prediction of 14-day mortality and validation

**Fig. 1:**
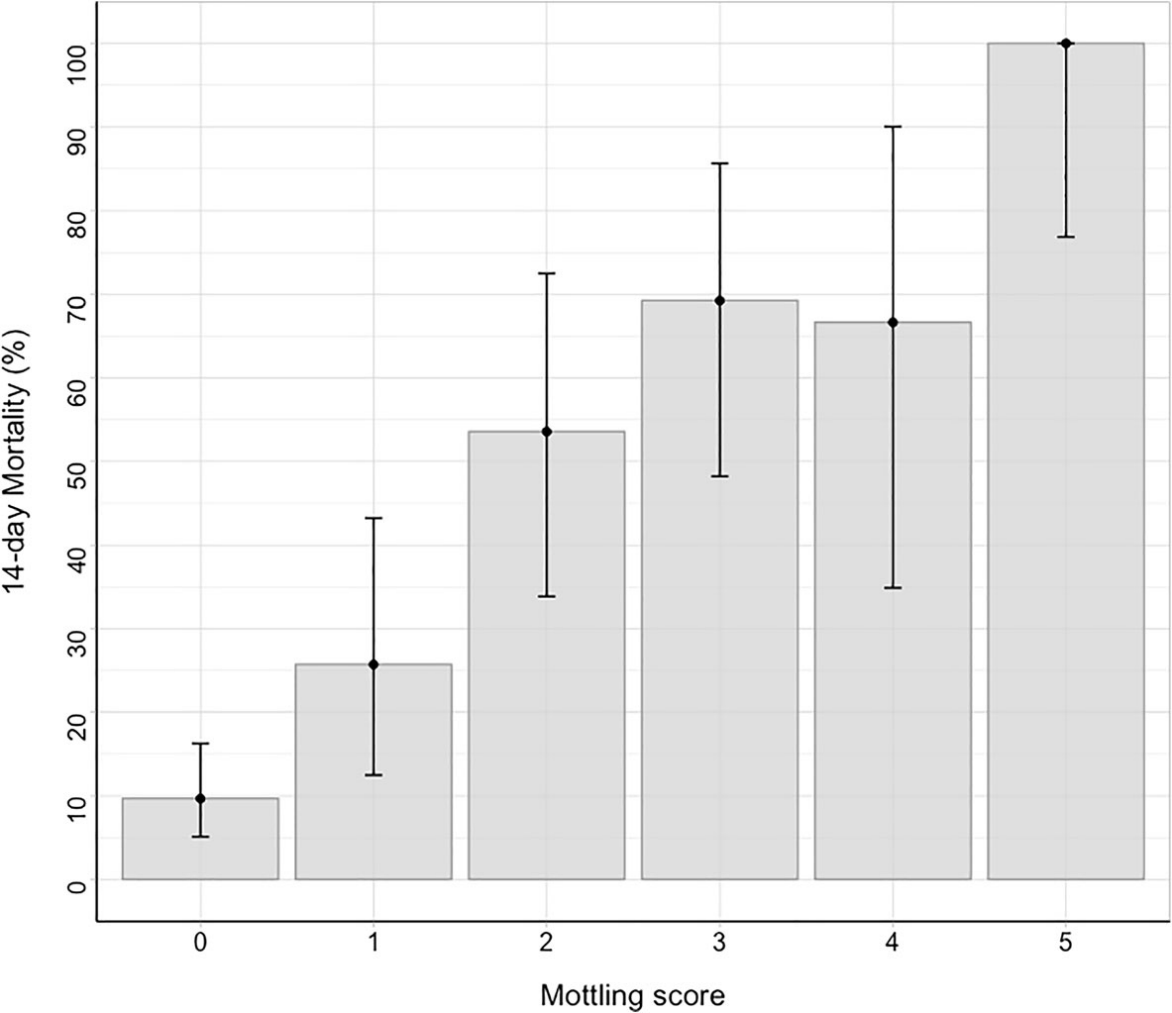
14-day mortality according to mottling score value at H-6. Error-bars represent 95% confidence interval

**Fig. 2:**
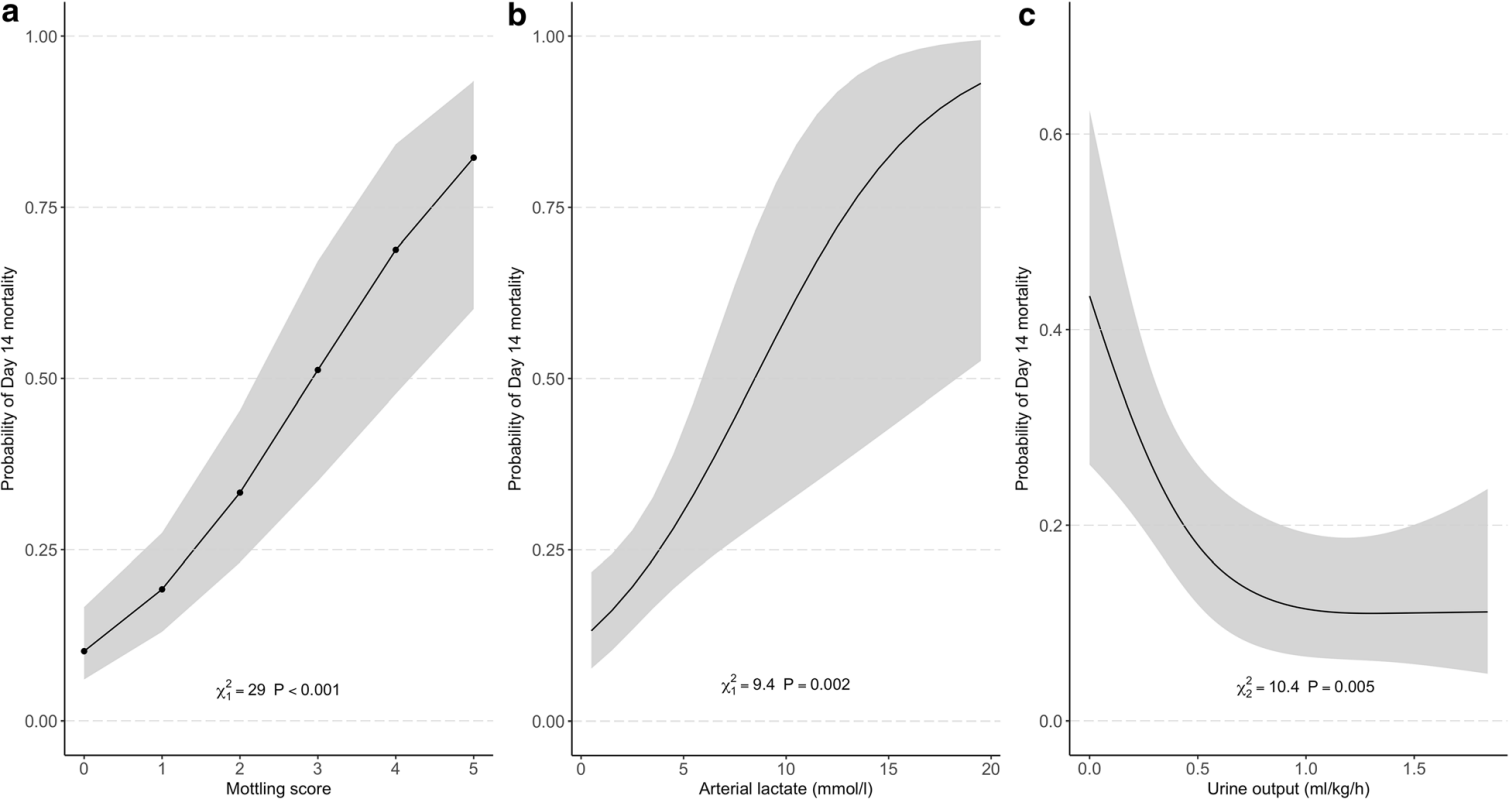
Adjusted effect of **a** mottling score, **b** arterial lactate, and **c** urine output at H6 on day 14 mortality (model 2). Results obtained by multivariable logistic regression with restricted cubic splines with three knots for urine output. Shaded area represents a 95% confidence interval for the trend

The 14-day mortality was 37% (95 deaths) in the development cohort and 38% (38 deaths) in the validation cohort. Figure 1 depicted the day-14 mortality according to the mottling score distribution at H6. In univariate analyses, several factors were associated with mortality (Additional file 1: Table S2): cirrhosis, SAPSII, pneumonia as the sepsis source, heart rate, urinary output, arterial lactate level, vasopressor requirement, vasopressor dose, and mottling score. In the multivariate analysis, three factors remained associated with day-14 mortality: mottling score (odds ratio (OR) 2.26, 95% CI 1.72–2.96, *p* value < 0.001), arterial lactate level (OR 1.29, 95% CI 1.11–1.50, *p* value < 0.001), and urine output < 0.5 ml/Kg/h (OR 3.03, 95% CI 1.37–6.71, *p* value 0.01) (model 1, Table 2). Results were slightly modified by using splines in model 2. Resulting plots depicting the effect of each predictor on 14-day mortality are displayed in Fig. 2. As shown, the effect of urine output on mortality tends to disappear at the threshold of 0.5 ml/kg/h.

**Table 2:** Factors associated with mortality at day 14 (multivariate analysis)

	Model 1	
Variables	OR [95% CI]	*P* value
Mottling score at H6, by point	2.26 [1.72–2.97]	< 0.001
Arterial lactate at H6, by 1 mmol/l	1.29 [1.11–1.50]	< 0.001
Urine output at H6 < 0.5 ml/kg/h	3.03 [1.37–6.69]	0.01
	Model 2	
Variables	OR [95% CI]*	*P* value
Mottling score at H6, by point	2.1 [1.60–2.75]	< 0.001
Arterial lactate at H6, by 1 mmol/l	1.26 [1.09–1.47]	0.002
Urine output at H6 (ml/kg/h)	–	0.005

*Odds ratio were not calculated for variables modeled using restricted cubic splines

Non-collinear variables included in the logistical regression model were mottling score at H6, arterial lactate at H6, urine output at H6, mean arterial pressure at H6, heart rate at H6, vasopressor dose at H6, and cirrhosis. In models 1 and 2, all variables were introduced continuously but urine output due to violation of the log-linearity assumption which was coded either as dichotomized according to some threshold in model 1, while as a spline function in model 2

C statistic of models 1 and 2 was 0.91 [0.87–0.95] and 0.89 [0.85–0.94], respectively. Using bootstrap validation, the optimism-corrected C statistic was 0.89 for model 1 and 0.88 for model 2, indicating the predictive ability of model in future patients. Applying our final prediction models to the validation cohort gave a C statistic of 0.76 [0.67–0.87] (model 1) and 0.75 [0.64–0.86] (model 2) with good calibration (Hosmer–Lemeshow test, *p* value = 0.69). C statistic was improved compared to that of SOFA, though not significantly in the validation cohort (Additional file 1: Table S5).

Details on models performance were given in Additional file 1: Figures S4 and S5.

### Subgroup analyses

#### Impact of vasopressor doses

**Fig. 3:**
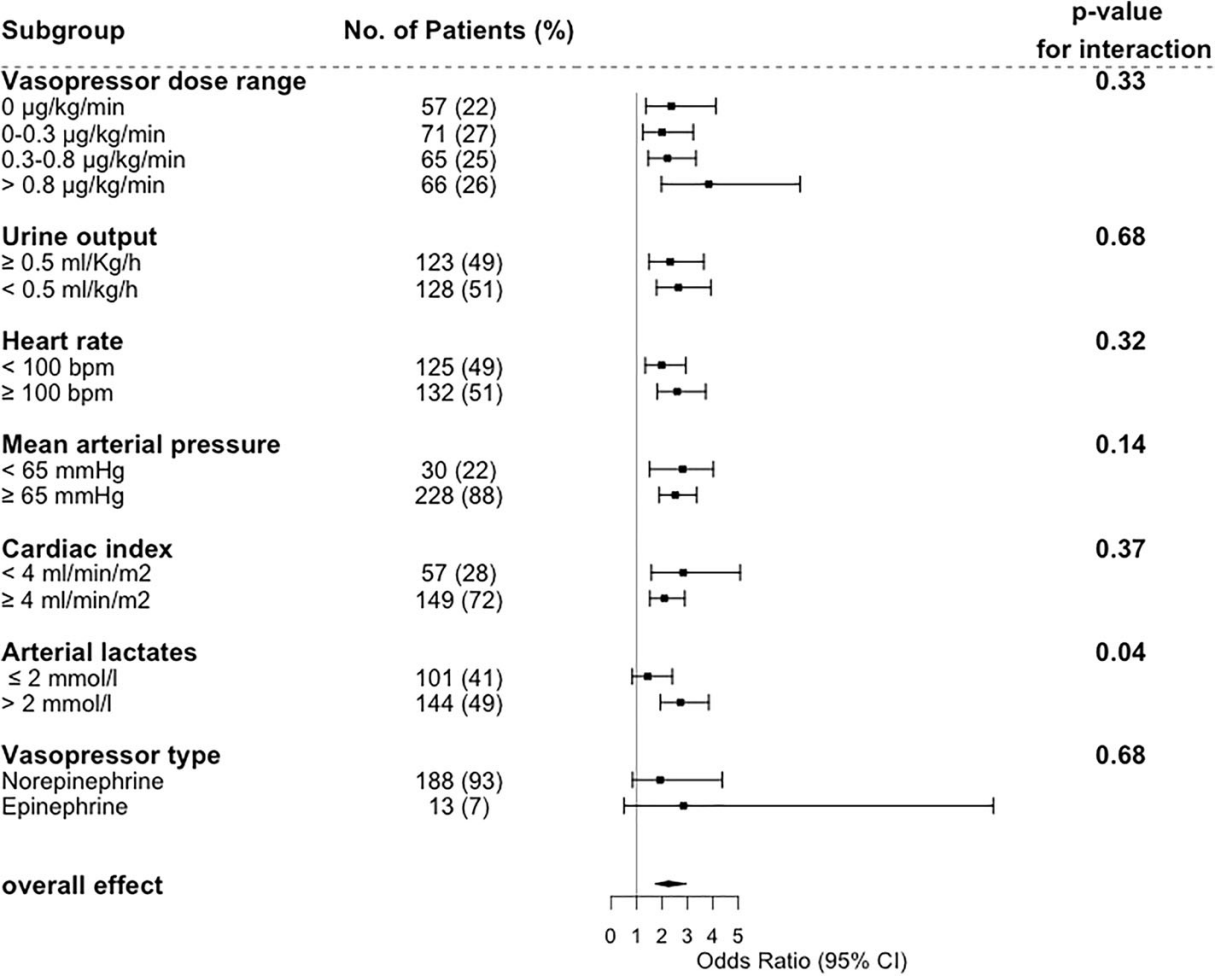
Effect of Mottling score at H6 on day 14 mortality according to different subgroups of patient. Estimates are Odds ratio with 95% confidence interval as estimated by logistic regression adjusted on SOFA score. Subset-by-Mottling score interactions on 14 day mortality were tested with the Gail and Simon test

First, we studied the effect of mottling score on 14-day mortality according to subgroups defined by vasopressor dose. As depicted in Fig. 3, OR ranged from 2.34 [1.10–3.15] in patients without vasopressor to 3.84 [1.98–7.43] in patients infused with vasopressors (> 0.8 μg/kg/min), without significant modification of the estimate (*p* value for interaction = 0.33); such an interaction with dose was similarly non-significant when dose was considered as a continuous parameter (*p* = 0.32). In other words, mottling score, as a marker of tissue hypoperfusion, predicts death whatever the vasopressor dosage. This is otherwise displayed in Additional file 1: Figure S6.

#### Interaction between mottling score and other perfusion parameters

Next, we investigated the effect of mottling score on mortality according to MAP, heart rate, arterial lactate level, and urine output (Fig. 3). We did not find any significant difference in mottling score effect across the subgroups, either based on urine output (*p* for interaction = 0.68), MAP (*p* for interaction = 0.14), heart rate (*p* for interaction = 0.32), or cardiac index (*p* for interaction = 0.37). By contrast, we found a quantitative interaction between the effect of mottling score on mortality and the arterial lactate level (*p* = 0.04).

#### Mottling score changes and mortality

A decrease of mottling score between the first 6 h of resuscitation was significantly associated with better outcome after adjustment on SOFA (*p* = 0.001). This result was confirmed whatever the mottling score at admission (Additional file 1: Figure S7).

#### Sensitivity analyses

Concerning the impact of mottling score on mortality, results were similar in the subgroup of patients with septic shock (*p* value for interaction = 0.69; Additional file 1: Figure S8), without any change according to the drug used (norepinephrine/epinephrine, Fig. 3 and Additional file 1: Table S3). One hundred thirty-seven (53%) patients fulfilled the SEPSIS 3 criteria for septic shock. Additional file 1: Table S4 reports the main characteristics of patients according to these criteria. As expected, patients with SEPSIS-3 definition have higher severity of illness (SOFA score 13 [10–16] vs. 7 [3–10]; *p* value< 0.01) and higher mottling score (2 [0–3] vs. 0 [0–1], *p* value < 0.001). Day-14 mortality was higher in this subset of patients (55% vs 16%; *p* < 0.01). However, the model performance to predict mortality was slightly affected (C statistic 0.94 [0.91–0.97] in the development cohort and 0.78 [0.63–0.93] in the validation cohort, Additional file 1: Table S5). Performances of the model to predict in-ICU mortality and in-hospital mortality are reported in Additional file 1: Table S5. Briefly, the model has a good performance to predict ICU mortality (development cohort: 106 deaths, C statistic 0.89 [0.84–0.94]; validation cohort: 62 deaths, C statistic 0.72 [0.62–0.81]).

Complete case analysis on 190 patients with no missing data (73 deaths) reached close findings, as detailed in the Additional file 1: Table S6.

## Discussion

We previously reported that the mottling score was associated with 14-day mortality in a small sample of septic shock patients. Here, in a larger cohort of patients with sepsis and septic shock, we found that the mottling score, measured 6 h after initial resuscitation, is a strong predictor of both 14-day and in-ICU mortality, death rate increasing linearly from stage 0 to stage 5. This result was validated in an independent cohort in another country and remained significant when using the recent SEPSIS-3 definition of septic shock. In addition, the mottling score predicted mortality whatever vasopressor dose and other hemodynamics parameters such as MAP or arterial lactate level. Moreover, mottling score improvement was significantly associated with better survival, whatever the patient severity at admission.

An important observation of the present study is the significant relationship between mottling score value and mortality. This effect remained after adjustment on patient severity and other well-known prognostic variables. It has been already suggested that microcirculation disorders were associated with outcome [4, 26]. However, techniques to explore microcirculation are complex [27] and not always available at bedside [28]. Mottling score is a very “easy to use easy to learn” non-invasive tool [14, 29], reflecting peripheral cutaneous tissue perfusion [30]. Our study confirms the important clinical relevance of mottling score and its quantitative value. In addition, we found that mottling improvement during resuscitation was associated with lower mortality.

Two others important predictors of mortality were identified in our study, namely lactate level and mean urine output at H6. Lactate level has been previously associated with outcome in septic patients [31, 32] together with its clearance during resuscitation [33–36]. However, an interesting finding in our study was the relationship between the prognostic effect of the mottling score and arterial lactate. Indeed, the effect of mottling score increased though not significantly with the level of arterial lactate, in line with Vellinga et al. who reported that hyperlactatemia was associated with sublingual microcirculation alterations [36]. Whether combining mottling score and arterial lactate during resuscitation in decision-making could be of interest to improve outcome prediction and potentially to more accurately guide therapy or not is an open issue for future studies. The recent randomized study by Hernandez et al. using repetitive measurements of capillary refill time suggests that peripheral perfusion-guided resuscitation of septic shock is a promising therapeutic approach [37].

Oliguria is a controversial predictor of outcome in ICU setting [38–40]. As a marker of organ perfusion-related dysfunction, it could be an interesting target during resuscitation. It has been found that oliguria may precede the increase of plasma creatinine and acute kidney injury development [41, 42]. However, mechanisms that regulate urinary output are complex. If oliguria seems to be related to acute kidney development [39, 43], there were conflicting data on its direct role on mortality [41, 44]. In addition, urine output as a goal of resuscitation therapy during sepsis has been no longer recommended in the last version of the “Surviving sepsis campaign” [45]. In the present study, we observed that oliguria is a strong predictor of death. We also found that early alteration of urine output (i.e., during the first 6 h of management) is associated with mortality. When we modeled urine output as a continuous variable, its effect on mortality increased at threshold of 0.5 ml/Kg/h, paving the way for future research.

It has been suggested that high doses of vasopressor influence patient outcome and increase microcirculation disorders [4, 46, 47]. However, the dose of vasopressors did not significantly modify the impact of mottling on 14-day mortality. In addition, we did not observe any significant variation in this effect according to MAP, heart rate, and cardiac index even after adjustment on patient severity. These observations sustained the loss of hemodynamic coherence described by several authors in sepsis context [10, 48].

Our study has several limitations. First, it was an observational study and we cannot rule that some heterogeneity was introduced in studied populations or procedures. However, general management and data collection were protocolized without great disparities. Nevertheless, mottling score remained an independent predictor of mortality in multivariate analysis, which controlled for several potential confounding factors. Second, dark skin patients have been excluded from analysis, because mottling could not be accurately assess. Other tools to evaluate skin tissue perfusion in patients with dark skin are currently under investigation such as infrared thermography [49] or laser doppler Imaging [50]. Third, it used data from 259 patients and our results need to be confirmed in a large population. However, these results were externally validated in an independent dataset, with good discrimination and calibration in both cohorts. We also performed internal validation using bootstrap resampling methods. As shown, our model has a reduced optimism suggesting a good ability of the model in future patients. The time point of interest, for the mottling score, was H6 though other measurements were performed (H0, H12, H24). We assume that this time point was clinically relevant because diagnosis investigations (including imaging) and both initial monitoring and resuscitation requires few hours. Last, we used 14-day mortality as the main outcome given long-term mortality may be influenced by comorbidities or ICU-acquired complications. Thus, it seems more relevant to explain directly sepsis-related mortality. However, in sensitivity analyses, the proposed model has good performance to predict in-ICU and in-hospital mortality.

## Conclusion

During sepsis and septic shock, mottling score reflecting peripheral tissue hypoperfusion was a strong predictor of mortality together with lactate level and urine output after initial resuscitation.

## Additional file

Additional file 1: **Table S1.** The main characteristics of patients included in the development and validation cohort. **Table S2.** Comparison of baseline and H6 characteristics according to mortality at day 14. **Table S3.** Factors associated with mortality at day 14 (multivariate analysis- restricted on 246 patients without epinephrine infusion). **Table S4.** Main characteristics of patients in the development cohort according to septic shock definition. **Table S5.** C statistic for prediction of different outcomes by final model. **Table S6.** Factors associated with mortality at day 14 on complete data (multivariate analysis). **Figure S1.** Flow chart of the study. **Figure S2.** Relationship between mottling score at H-6 and 14-day mortality. **Figure S3.** Effect of vasopressor dose at H-6 on day-14 mortality (univariate analysis). **Figure S4.** Calibration plot (model 1 and 2). **Figure S5.** Discrimination of the model assessed using the C statistic. **Figure S6.** Observed and predicted probabilities of death by mottling score according to range of vasopressor dose (adjusted on SOFA score). **Figure S7.** Effect of Mottling score decrease between H0 and H6 on day 14 mortality. **Figure S8.** Effect of mottling score by vasopressor dose at H6. (DOCX 19921 kb)

## Abbreviations

HR: Heart rate
ICU: Intensive care unit
IQR: Interquartile range
MAP: Mean arterial pressure
OR: Odds ratio
SAPS II: Simplified acute physiologic score II
SOFA: Sequential organ failure assessment
