# Supplementary material for: Mottling score is a strong predictor of 14-day mortality in septic patients whatever vasopressor doses and other tissue perfusion parameters
Source: Crit Care. 2019 Jun 10;23:211. doi: 10.1186/s13054-019-2496-4 (PMC6558704; doi:10.1186/s13054-019-2496-4)
Supplement: Supplementary file 1 — Table S1. The main characteristics of patients included in the development and validation cohort. Table S2. Comparison of baseline and H6 characteristics according to mortality at day 14. Table S3. Factors associated with mortality at day 14 (multivariate analysis- restricted on 246 patients without epinephrine infusion). Table S4. Main characteristics of patients in the development cohort according to septic shock definition. Table S5. C statistic for prediction of different outcomes by final model. Table S6. Factors associated with mortality at day 14 on complete data (multivariate analysis). Figure S1. Flow chart of the study. Figure S2. Relationship between mottling score at H-6 and 14-day mortality. Figure S3. Effect of vasopressor dose at H-6 on day-14 mortality (univariate analysis). Figure S4. Calibration plot (model 1 and 2). Figure S5. Discrimination of the model assessed using the C statistic. Figure S6. Observed and predicted probabilities of death by mottling score according to range of vasopressor dose (adjusted on SOFA score). Figure S7. Effect of Mottling score decrease between H0 and H6 on day 14 mortality. Figure S8. Effect of mottling score by vasopressor dose at H6. (DOCX 19921 kb) [file 13054_2019_2496_MOESM1_ESM.docx]

**Mottling score is a strong predictor of 14-day mortality in septic patients whatever vasopressor doses and other tissue perfusion parameters.**

Guillaume Dumas, MD; Jean-Rémi Lavillegrand, MD, Jérémie Joffre, MD.Ph.D, Naïke Bigé, MD.Ph.D; Edmilson Bastos de-Moura, MD; Jean-Luc Baudel, MD; Sylvie Chevret, MD.Ph.D; Bertrand Guidet, MD.Ph.D, Eric Maury, MD.Ph.D; Fabio Amorim, MD; Hafid Ait-Oufella, MD.Ph.D.

**Additional file 1**

**Statistical Methods**

*Restricted Cubic Splines*

In the present study, restricted cubic splines (RCS) were used to characterize the relationship of mottling score, vasopressor dose, urine output, mean arterial pressure and heart rate with the outcome. Restricted cubic splines allow assessing the relationship between continuous independent variables with the outcome, relaxing any prior assumption regarding the shape of this relationship (while standard models assume log-linearity). We address the potential nonlinearity of the association using Wald test where the null-hypothesis was a linear effect of covariate on the outcome.

These piecewise polynomials functions satisfy continuity constraints at the knots joining the pieces. The number of knots was based on Akaike Information Criterion (AIC). In the regression spline model (model 2), the Wald test for the regression coefficients was used to test the null hypothesis that the variable as no effect.

**Table S1: Main characteristics of patients included in development and validation cohort**

|  | **Development cohort**  **N= 259** | **Validation cohort**  **N=97** | **Standardized**  **mean difference** |
| --- | --- | --- | --- |
| Age, year | 68 [57-80] | 77 [69-84] | 0.34 |
| Female sex | 126 (49) | 55 (57) | 0.16 |
| Weight, kg | 70 [60-81.75] | 70 [60-80] | 0.049 |
| SAPS II score, points | 54 [41-70] | 46 [36-57] | 0.51 |
| SOFA score at day 1, points | 10 [5-14] | 9 [8-11] | 0.19 |
| **Pre-existing conditions** |  |  |  |
| Atherosclerotic disease | 67 (26) | 6 (6) | 0.56 |
| Cirrhosis | 27 (12) | 2 (2.1) | 0.62 |
| **Sepsis origin** |  |  | 0.59 |
| Community-acquired pneumonia | 113 (44) | 65 (67) |  |
| Intra-abdominal | 65 (26) | 18 (19) |  |
| Urinary tract | 28 (11) | 9 (9) |  |
| Skin and soft tissue | 21 (8) | 1 (1) |  |
| Other | 27 (11) | 4 (4) |  |
| **Clinical parameters** |  |  |  |
| Mean arterial pressure, mm Hg | 73 [67-82] | 83 [58-90] | 0.52 |
| Urinary output, ml/kg/h | 0.46 [0.13-0.90] | 0.95 [0.375-1.5] | 0.53 |
| Arterial lactate, mmol/l | 2.4 [1.4- 5.3] | 1.7 [1.2-3.0] | 0.48 |
| Mottling score |  |  | 0.545 |
| 0 | 125 (49) | 59 (61) |  |
| 1 | 36 (14) | 21 (22) |  |
| 2 | 29 (11) | 8 (8) |  |
| 3 | 29 (11) | 5 (5) |  |
| 4 | 14 (5) | 3 (3) |  |
| 5 | 24 (9) | 1 (1) |  |
| **Drugs** |  |  |  |
| Norepinephrine | 188 (73) | 97 (100) | 0.86 |
| Doses, μg/kg/min | 0.5 [0.2-1] | 0.3 [0.13-0.6] | 0.06 |
| Epinephrine | 13 (5) | - | - |
| Doses, μg/kg/min | 0.6 [0.3-1] | - |  |
| Dobutamine | 4 (2) | - | - |
| Doses, μg/kg/min | 0.3 [0.18-1.3] | - |  |
| **Mortality at day 14** | 95 (37) | 37 (38) | 0.030 |

Results are given as N (%) or Median [IQR]

SAPS II: Simplified Acute Physiology Score was calculated within 24 h of intensive care unit admission. SOFA: Sequential Organ Failure Assessment score was calculated within 24 hour of septic shock onset. MAP: Mean arterial pressure.

**Table S2.** Comparison of baseline and H6 characteristics according to mortality at Day 14

| **Demographic and Clinical Data** | **Survivors**  **(n= 164)** | **Non survivors**  **(n= 95)** | **P**  **value** |
| --- | --- | --- | --- |
| **Characteristics of the patients** |  |  |  |
| Age, year | 67 [56-80] | 70 [60-81] | 0.20 |
| Male sex | 92 (56) | 41 (43) | 0.06 |
| Vascular diseases | 123 (75) | 68 (72) | 0.75 |
| Cirrhosis | 10 (7) | 17 (20) | 0.007 |
| SAPS II, points | 47 [37-60] | 71 [55-89] | <0.0001 |
| Community-acquired pneumonia | 82 (51) | 59 (64) | 0.05 |
| **Clinical parameters at H6** |  |  |  |
| Mottling score | 0 [0-1] | 3 [2-5] | <0.0001 |
| Heart rate,  > 100 beats/min | 66 (40) | 61 (65) | 0.0003 |
| Mean arterial pressure,  < 65 mmHg | 15 (9) | 15 (16) | 0.16 |
| Cardiac index,  < 4 l/min/m^2^ | 33 (24) | 24 (35) | 0.15 |
| Urinary output,  < 0.5 ml/kg/h | 57 (35) | 71 (79) | <0.0001 |
| Arterial lactate, mmol/l | 1.9 [1.2-2.8] | 6.06 [2.65-12.05] | <0.0001 |
| **Drugs at H6** |  |  |  |
| Vasopressor at H-6 | 115 (71) | 85 (89) | <0.0008 |
| Dose of vasopressor, μg/kg/min | 0.2 [0-0.4] | 0.7 [0.3-1.25] | <0.0001 |
| **Mechanical ventilation at day 1** | 91 (57) | 36 (61) | 0.66 |

Results are given as N (%) or Median [IQR]. P-value were calculated as Chi_2 or Wilcoxon tests.

SAPS II: Simplified Acute Physiology Score was calculated within 24 h of intensive care unit admission.

**Figures**

**Figure S1. Flow chart of the study**

**
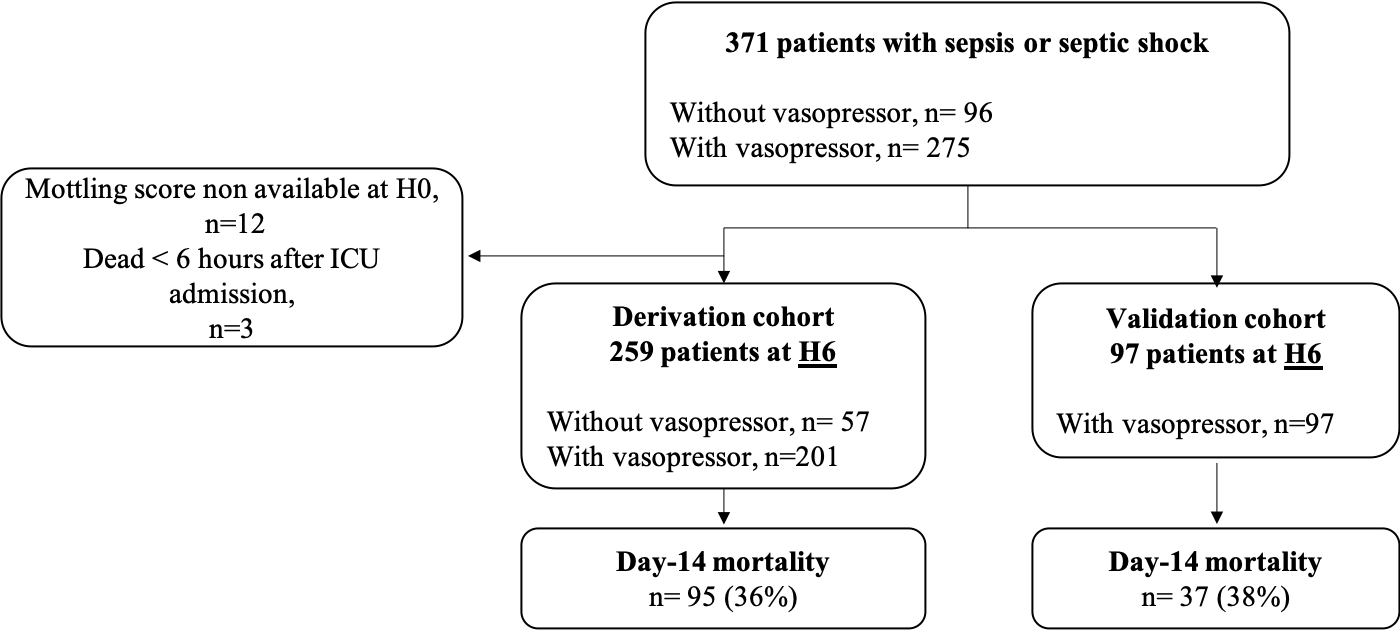
**

**Figure S2. Relationship between mottling score at H-6 and 14-day mortality**

**
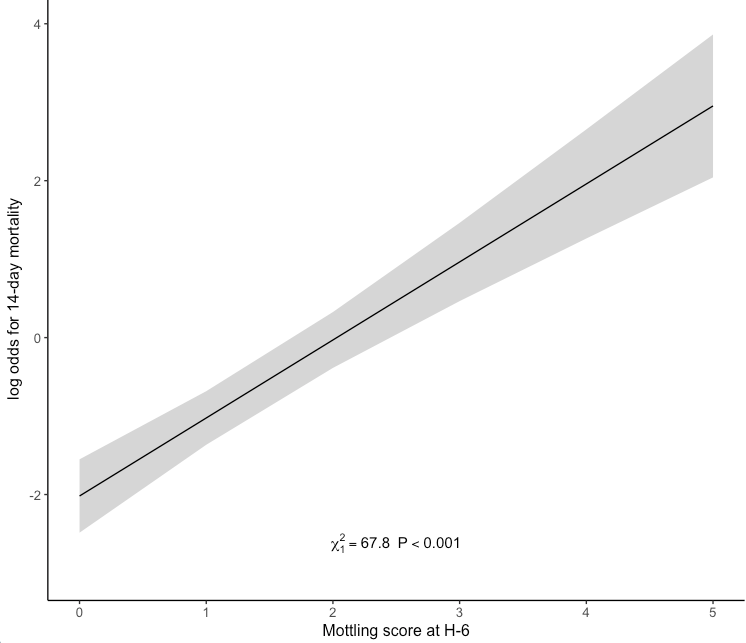
**

Plot depicts relationship between predictor and mortality. The solid line indicates effect estimate and shaded area the 95 percent confidence intervals. P value for association was given with Wald test. As shown, there was a linear relationship between predictor and outcome (p value for non-linearity: p=0.61, Wald test).

**Figure S-3. Effect of vasopressor dose at H-6 on day-14 mortality (univariate analysis)**


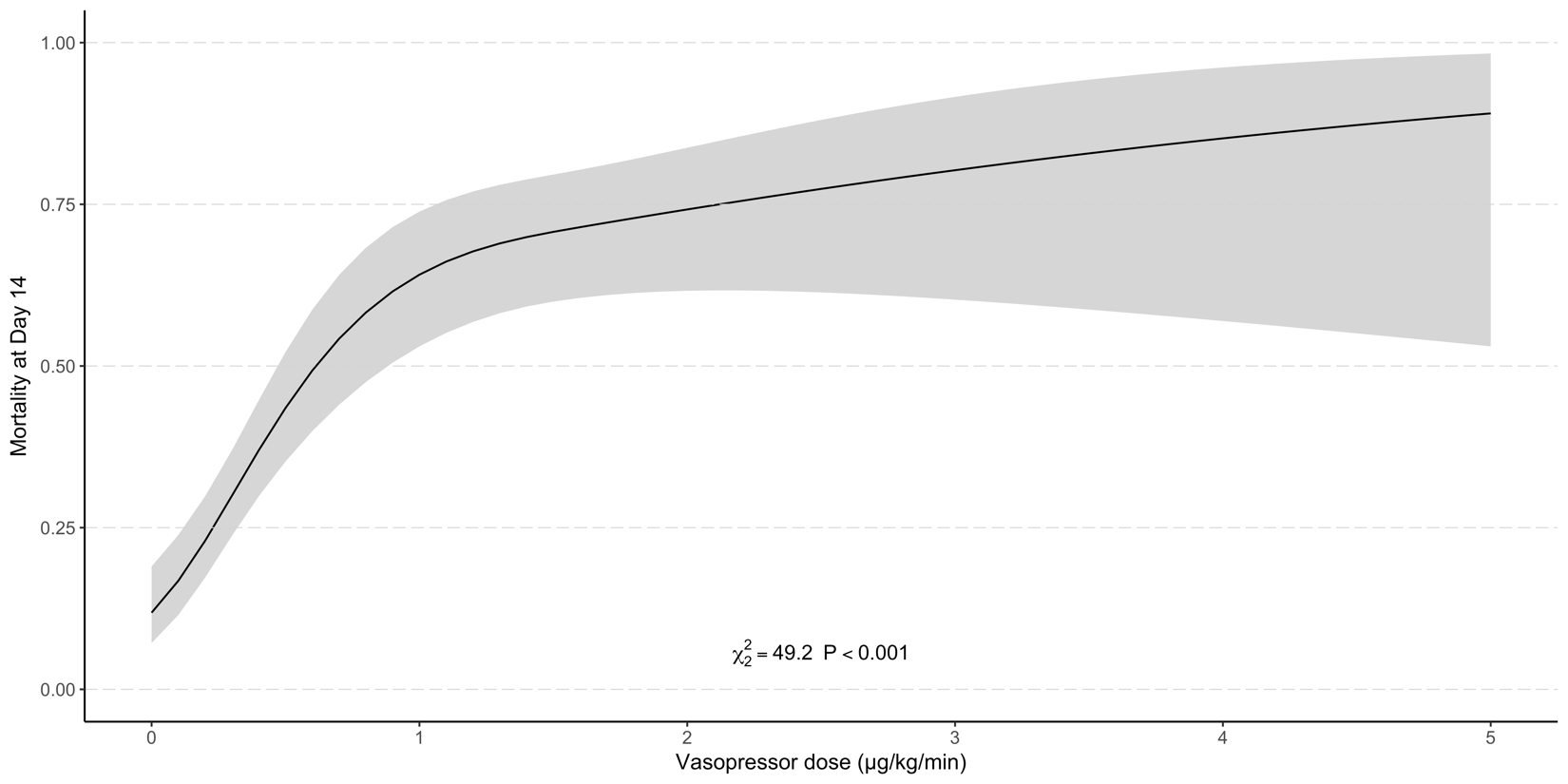


The probability of day-14 mortality (solid line) and 95 percent confidence intervals (shaded area) were estimated by restricted cubic-spline logistic regression analysis using 3 knots at 0.10^th^, 0.5^th^ and 90^th^.


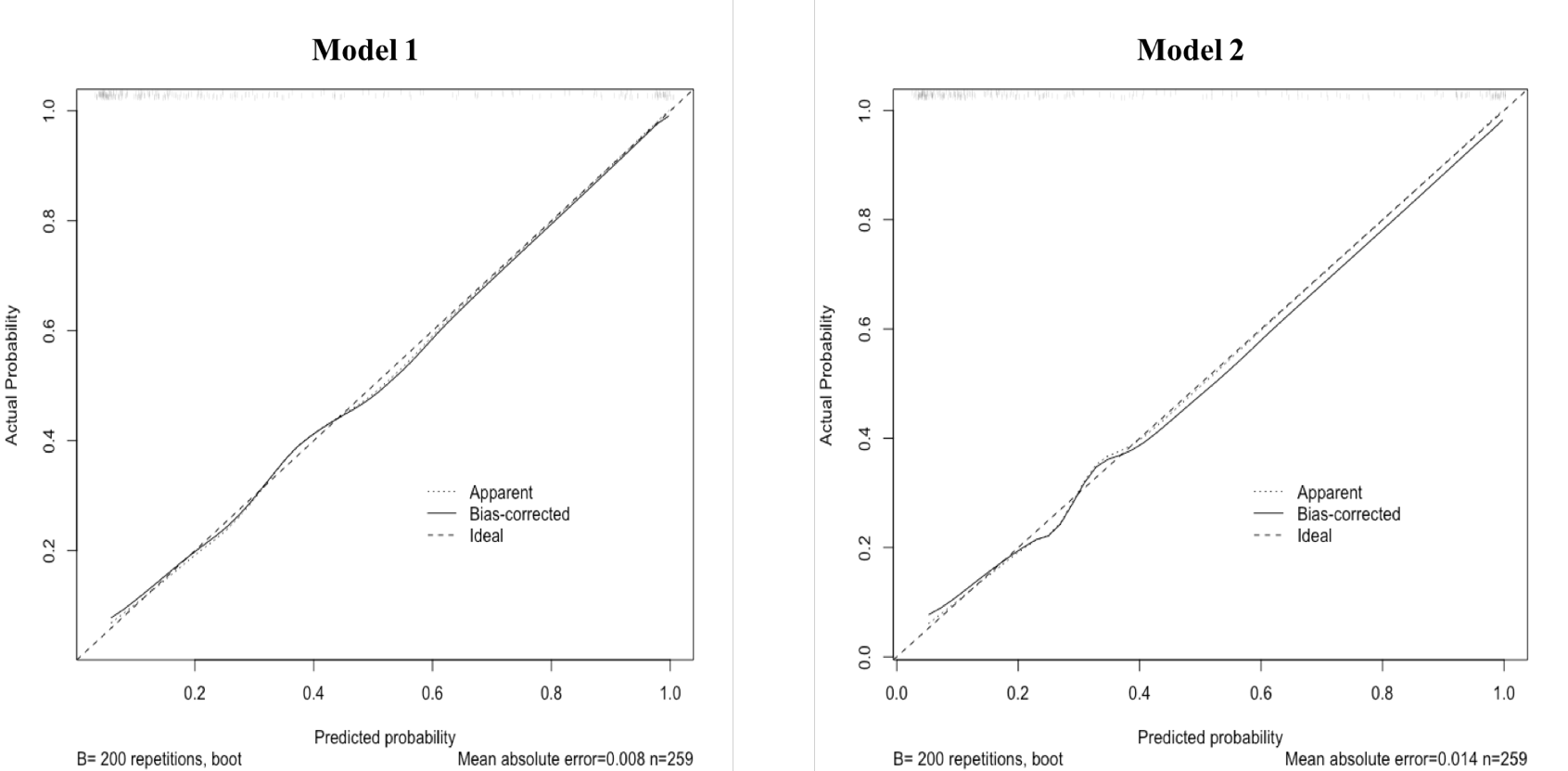
These plots compare observed and predicted probability of death according to model 1 and 2. The apparent curve (dashed line) represent the result of the study. Bootstrap methods (200 repetitions) were used to obtain the bias-corrected curve for internal validation (solid line). The 45-degree line describes the perfect prediction (dotted line). The closeness of the calibration curve to the 45^◦^ line demonstrates excellent validation.

**Figure S-4. Calibration plot (model 1 and 2)**

**Figure S5. Discrimination of the model assessed using the C statistic**


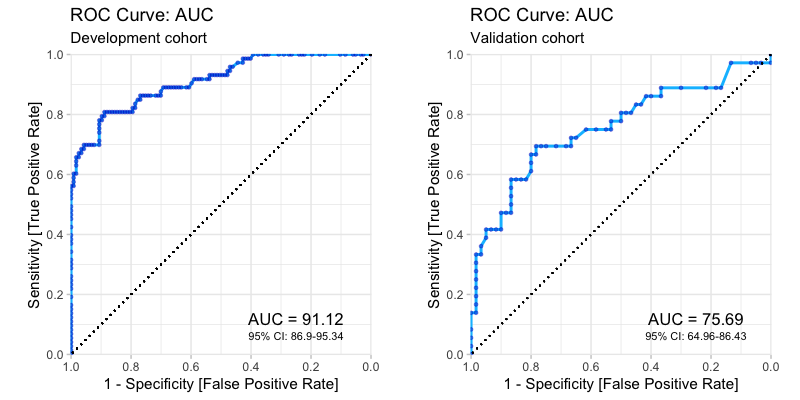


**Figure S6. Observed and predicted probabilities of death by mottling score according to range of vasopressor dose (adjusted on SOFA score)**


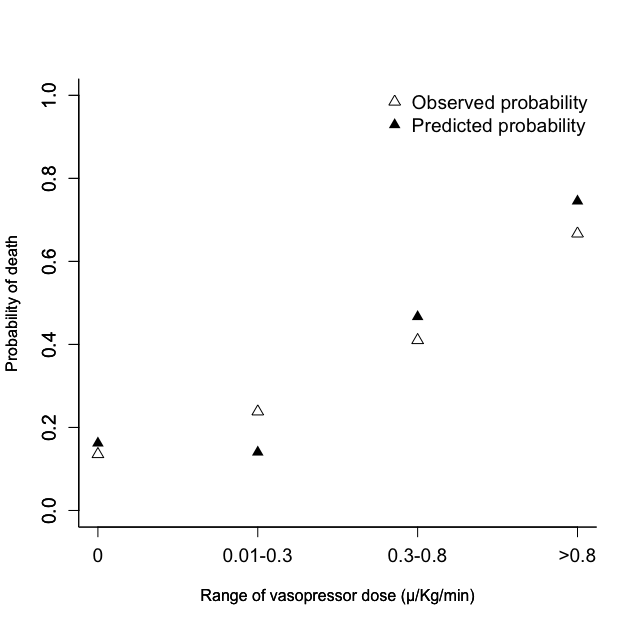


**Figure S7. Effect of Mottling score decrease between H0 and H6 on day-14 mortality**


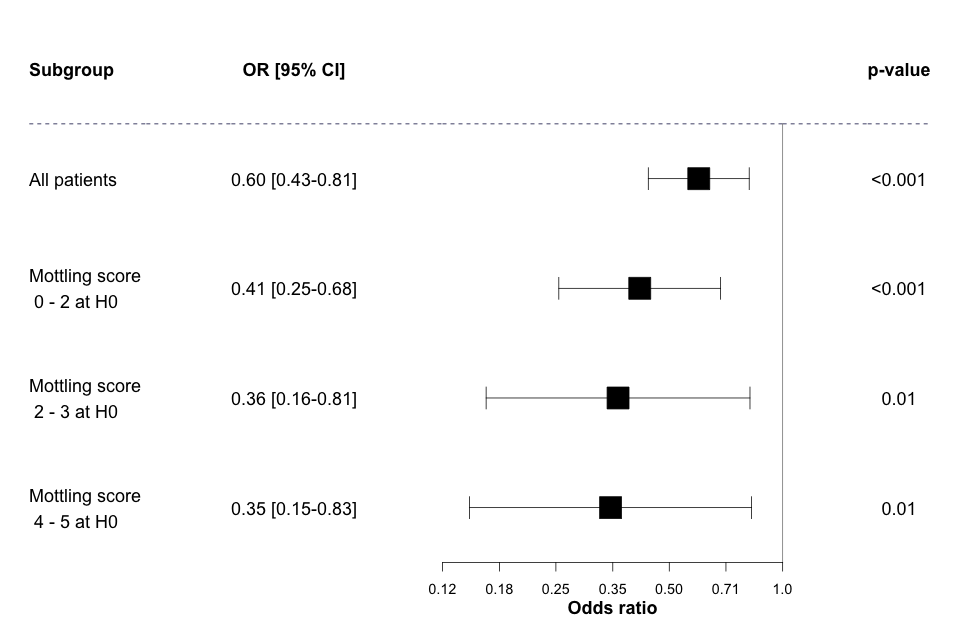


Estimates are odds ratio with 95 confidence intervals as estimated by logistic regression adjusted on SOFA score.

**Sensitivities analysis**

**1- Effect of mottling score according to vasopressor dose at H6 (analysis restrained on 202 patients with septic shock)**

**Figure S-8. Effect of mottling score by vasopressor dose at H6.**


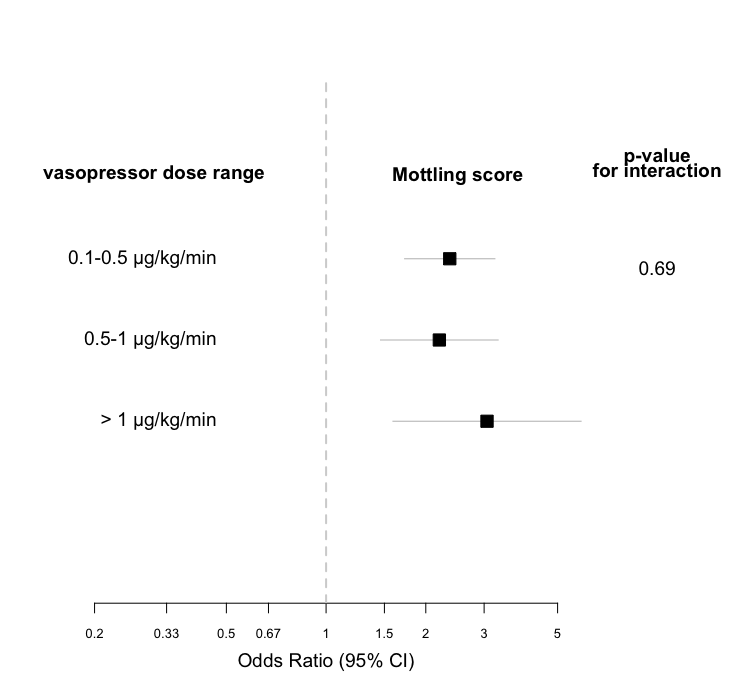


Estimates are Odds ratio and 95% confidence interval as estimated by logistic regression adjusted on SOFA score. Vasopressor range were quantile of dose at H6.

Effect of mottling score by vasopressor range interaction was based on the Gail and Simon statistic.

**2- Analysis restrained to patients without epinephrine infusion**

**Table S3.** Factors associated with mortality at day 14 (multivariate analysis- restricted on 246 patients without epinephrine infusion)

|  | **Model 1** | | |
| --- | --- | --- | --- |
| **variable** | | **OR [95% CI]** | **P**  **value** |
| **Mottling score at H6, by point** | | 4.75 [2.72 – 8.28] | <0.001 |
| **Arterial lactate at H6, by 1 mmol/l** | | 1.29 [1.41 – 4.16] | 0.001 |
| **Urine output at H6 <0.5 ml/kg/h** | | 3.03 [1.287 – 6.41] | 0.01 |
|  | **Model 2** | | |
| **variable** | | **OR [95% CI]^a^** | **P**  **value** |
| **Mottling score at H6, by point** | | 4.26 [2.46 – 7.39] | <0.001 |
| **Arterial lactate at H6, by 1 mmol/l** | | 2.29 [1.32 -3.96] | 0.003 |
| **Urine output at H6 (ml/kg/h)** | | - | 0.01 |

**Odds ratio were not calculated for variables modeled using restricted cubic splines*

**3-Anaysis restrained on various assumptions and subset of patients**

- **Patients with SEPSIS-3 definition of septic shock**

**Table S4.** Main characteristics of patients in the development cohort according to septic shock definition

| **Demographic and Clinical Data** | **Old sepsis definition**  **(n= 122)** | **Sepsis-3 definition**  **(n= 137)** | **P**  **value** |
| --- | --- | --- | --- |
| **Characteristics of the patients** |  |  |  |
| Age, year | 70 [59-77] | 68 [56-81] | 0.98 |
| Vascular diseases | 35 (29) | 32 (23) | 0.38 |
| SOFA score, points | 7 [3-10] | 13 [10-16] | <0.01 |
| SAPS II, points | 45 [35-57] | 65 [50-80] | <0.01 |
| Community-acquired pneumonia | 57 (48) | 56 (41) | 0.31 |
| **Clinical parameters at H6** |  |  |  |
| Mottling score, (min-max) | 0 [0-1]; (0-5) | 2 [0-3]; (0-5) | <0.01 |
| Heart rate,  > 100 beats/min | 39 (32) | 88 (64) | <0.01 |
| Mean arterial pressure,  < 65 mmHg | 13 (11) | 17 (12) | 0.82 |
| Cardiac index,  < 4 l/min/m^2^ | 29 (29) | 28 (26) | 0.73 |
| Urinary output,  < 0.5 ml/kg/h | 31 (26) | 97 (74) | <0.01 |
| Arterial lactate, mmol/l | 1.4 [0.9-1.8] | 4.9 [2.7-9.4] | <0.01 |
| **Drugs at H6** |  |  |  |
| Vasopressor at H-6 | 64 (53) | 137 (100) | <0.01 |
| Dose of vasopressor, μg/kg/min | 0.5 [0-0.25] | 0.6 [0.3-1.2] | <0.01 |
| **Mechanical ventilation at day 1** | 67 (56) | 60 (60) | 0.63 |
| **Day-14 mortality** | 20 (16) | 75 (55) | <0.01 |

- **Model performance on different assumptions**

**Table S5.** C-statistic for prediction of different outcomes by the proposed model.

|  | **Development cohort** | | **Validation cohort** | |
| --- | --- | --- | --- | --- |
|  | **No. of death/No. of patients (%)** | **C-statistic**  **[95%CI]** | **No. of death/ No. of Patients (%)** | **C-statistic**  **[95%CI]** |
| **day-14 mortality** | | | | |
| Proposed model on the entire cohort | 95/259 (37) | 0.91 [0.87-0.95] | 37/97 (38) | 0.76 [0.64-0.86] |
| Proposed model on patients fulfilled SEPSIS-3 definition* | 75/137 (55) | 0.94 [0.91-0.97] | 20/41 (49) | 0.78 [0.63-0.93] |
| SOFA score prediction |  | 0.81 [0.76-0.87] |  | 0.69 [0.58-0.80] |
| **ICU mortality** | | | | |
| Proposed model on the entire cohort | 106/259 (41) | 0.89 [0.84-0.94] | 62/97 (64) | 0.72 [0.62-0.81] |
| **Hospital mortality** | | | | |
| Proposed model on the entire cohort | 125/259 (48) | 0.84 [0.79-0.90] | NA | NA |

ICU : intensive care unit. SOFA : Sequential Organ Failure Assessment score.

**defined as the need for vasopressor and arterial lactates >2 mmol/l*

**4- Complete Data analysis (free of imputation)**

**Table S6.** Factors associated with mortality at day 14 on complete data (multivariate analysis)

**Model 1**

|  | **OR [95% CI]** | **P** **value** |
| --- | --- | --- |
| **Mottling score at H6, by point** | 2.07 [1.51-2.84] | <0.0001 |
| **Arterial lactate at H6, by 1 mmol/l** | 1.42 [1.17-1.73] | 0.0005 |
| **Urine output at H6 <0.5 ml/kg/h** | 2.68 [1.04-6.91] | 0.04 |

Non-collinear variables included in the logistical regression model were: mottling score at H6, arterial lactate at H6, urine output at H6, cirrhosis, mean arterial pressure at H6, heart rate at H6, vasopressor dose at H6. C-statistic of model: 0.91, Hosmer-Lemeshow p-value= 0.52

**Model 2**

|  | **OR [95% CI]*** | **P** **value** |
| --- | --- | --- |
| **Mottling score at H6, by point** | 1.92 [1.41 – 2.62] | <0.0001 |
| **Arterial lactate at H6, by 1 mmol/l** | 1.40 [1.15 -1.71] | 0.0008 |
| **Urine output at H6 (ml/kg/h)** | - | 0.05 |

**Odds ratio were not calculated for variables modeled using restricted cubic splines.*

Non-collinear variables included in the logistical regression model were: mottling score at H6, arterial lactate at H6, urine output at H6, mean arterial pressure at H6, heart rate at H6, vasopressor dose at H6, cirrhosis.

C-statistic of model: 0.90, Hosmer-Lemeshow p-value= 0.54
